# Glucose absorption following gastric and small intestinal nutrient administration in the critically ill

**DOI:** 10.1186/cc9807

**Published:** 2011-03-11

**Authors:** M Chapman, A Deane, A Di Bartolemeo, A Zaknic, M Summers, N Nguyen, L Besanko, C Burgstad, M Horowitz

**Affiliations:** 1 Royal Adelaide Hospital, Adelaide, Australia

## Introduction

Glucose absorption from the stomach is abnormal related to slow gastric emptying and impaired in critically ill patients (CIP) with normal gastric emptying, suggesting that small intestinal (SI) factors may also be responsible. Small intestinal absorption of nutrient has not been formally quantified in this group. The aim was to quantify and compare glucose absorption following gastric and SI administration in CIP and healthy volunteers (HV).

## Methods

Data from studies where glucose absorption had been measured were analysed. Sixty-six CIP (age: 51 ± 2, APACHE II: 17 ± 1) and 50 HV (age: 43 ± 3) were administered 100 ml Ensure (liquid nutrient 1.06 kcal/ml), labelled with 3 g 3-*O*-methylglucose (3-OMG) to evaluate glucose absorption. Nutrient was administered via nasogastric (*n *= 44; CIP = 24; HV = 20) or SI (*n *= 72; CIP = 42; HV = 30) catheters. Plasma 3-OMG concentrations were measured at intervals for 240 minutes; peak, time to peak and area under the concentration curve (AUC) were calculated. Feed-intolerant patients were defined by gastric residual volume >250 ml in the 24 hours prior to study or requiring prokinetics for pre-existing feed intolerance. Data are mean ± SEM or median (range) and were analysed using nonpaired Student's *t *tests.

## Results

Glucose absorption was markedly reduced in patients following both intragastric (AUC 0 to 240: CIP: 49 ± 7 vs. HV: 80 ± 4 mmol/l/minute; *P *< 0.001; peak concentration CIP: 0.32 (0.004 to 0.804) vs. HV: 0.51 (0.343 to 0.679) mmol/l; *P *< 0.001; time to peak CIP: 140 (30 to 240) vs. HV: 74 (45 to 120) minutes; *P *< 0.001) and SI nutrient (AUC 0 to 240: CIP: 57 ± 4 vs. HV: 72 ± 4 mmol/l/minute; *P *= 0.008; peak concentration CIP: 0.37 (0.01 to 0.88) vs. HV: 0.47 (0.28 to 0.88) mmol/l; *P *= 0.02; time to peak CIP: 87 (15 to 240) vs. HV: 54 (15 to 120) min; *P *= 0.01). Gastric glucose absorption was delayed when compared with SI administration in CIP (time to peak; gastric: 140 (30 to 240) vs. SI: 86 (15 to 240) minutes; *P *= 0.005); however, there was no difference in overall glucose absorption when comparing gastric and SI administration in both HV and CIP. Feed-intolerant patients had reduced SI glucose absorption (AUC 240: intolerant 44 (2 to 98) vs. tolerant 75 (15 to 101) mmol/l; *P *= 0.01).

## Conclusions

Glucose absorption is substantially impaired in the CIP even when delivered directly into the SI. This suggests mechanisms in the SI contribute to nutrient malabsorption. Delivery of nutrient directly into the SI (particularly in those CIP who are feed intolerant) may not result in improved nutrient absorption.

